# Antioxidant and Rutin Content Analysis of Leaves of the Common Buckwheat (*Fagopyrum esculentum* Moench) Grown in the United Kingdom: A Case Study

**DOI:** 10.3390/antiox8060160

**Published:** 2019-06-03

**Authors:** Solomon Habtemariam

**Affiliations:** Pharmacognosy Research Laboratories & Herbal Analysis Services UK, University of Greenwich, Chatham-Maritime, Kent ME4 4TB, UK; s.habtemariam@herbalanalysis.co.uk; Tel.: +44-208-331-8302

**Keywords:** buckwheat leaves, HPLC, antioxidant, fagopyrins, phototoxicity, functional foods

## Abstract

The common buckwheat, *Fagopyrum esculentum* Moench (Polygonaceae) is a gluten-free pseudocereal that has been gaining in popularity in recent years as a low-calorie and nutrient-rich healthy food option. Buckwheat farming is common in Eastern European countries and the Far East, while in the UK and other Western European countries, the plant has limited medicinal or food applications. The vegetative parts, particularly the leaves and flowers, are among the best-known sources of the bioactive compound, rutin. Hence, functional foods originated from buckwheat leaves are common, although the scope of such applications is limited by phototoxicity associated with the fagopyrin composition. Here, the antioxidant and rutin composition of the leaves of the plant grown in the UK are assessed. The methanol extract of the leaves displayed a potent DPPH (2,2-diphenyl-1-picrylhydrazyl) radical scavenging effect along with reducing power. Quantitative High Performance Liquid Chromatography (HPLC)-based analysis showed the rutin content of the leaves as 3417 mg/100g (on dry weight (DW) basis). The identity of rutin was also confirmed by isolation and structural elucidation based on spectroscopic studies. From the chemical content analysis, including fagopyrin levels and the antioxidant assays, UK-grown buckwheat has potential as a commercial source of rutin or as a functional food.

## 1. Introduction

**Figure 1:**
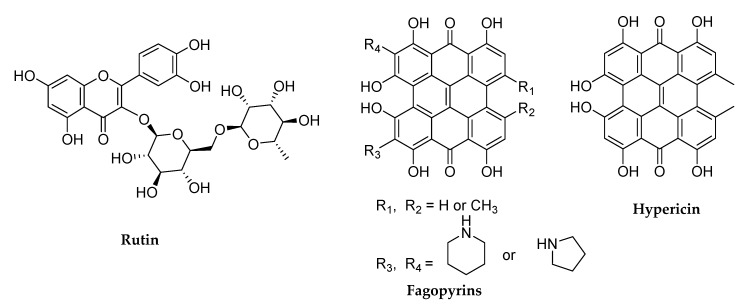
The structures of rutin, fagopyrins and hypericin. The phototoxicity of buckwheat green is known to attribute to the fagopyrins’ composition. These structures are similar with hypericin and differ from each other (for example, fargopyrins A–F) on the substituents of R_1_-R_4_ as shown in the figure.

The common buckwheat, *Fagopyrum esculentum* Moench (Polygonaceae), is a pseudocereal that contains the bioactive compound rutin (Figure 1) in high yield. Rutin, along with related bioflavonoids in the seeds, have been shown to have numerous pharmacological activities that would give buckwheat seeds the acclaimed health benefits [1]. The proteins in buckwheat seeds have also been claimed to have numerous health benefits, including hypocholesterolaemic, anti-inflammatory, and antioxidant effects, suppressing gallstones and tumors, and inhibiting the angiotensin I-converting enzyme [1,2,3]. Other health benefits of buckwheat seeds that attribute to their carbohydrate, proteins, fibres, and other macromolecular composition have also been well documented [3]. In view of the macromolecular components including starch and proteins, the potential utilisation of buckwheat both as a gluten-free diet in human consumption and as animal fodder have been advocated [4,5,6,7,8].

With respect to functional food development, the various parts of the common buckwheat plant including the pseudocereal, leaves, flowers, and sprouts have been extensively investigated in recent years [9,10,11]. These studies highlighted the therapeutic implication of the high polyphenol content in the plant which is dominated by rutin. In this regard, the leaves and flowers of the plant are known for the highest rutin content, though they also contain potentially phototoxic naphthodianthrone-based alkaloids, known as fagopyrins (Figure 1) [12]. Buckwheat leaves and flowers have also long been used as valuable sources for industrial production of rutin [13]. Large scale cultivation of the common buckwheat is mainly for its pseudocereal and extends from temperate Europe to Japan through the Indo-Myanmar region. Its adaptation to colder and European climates is mainly due to its frost resistance, its fast rate of growth and the rather little care required during its cultivation. Hence, buckwheat cultivation is common even in high-altitude regions such as in Tibet, growing at up to 4500 m above sea level. According to the Food and Agriculture Organization of the United Nations FAO, [14]), the top three producers of buckwheat in the world are the Russian Federation, followed by China and Ukraine (Table 1). In Europe, however, the once important source of food from buckwheat farming has been largely replaced by cereals such as wheat. In countries like the UK, buckwheat is in fact not farmed for its cereal but as a crop cover in limited capacity: for example, to enrich soil such as green manure, suppress weeds and attract bees and other insect pollinators. By assessing the antioxidant effect and rutin content of the leaves, this case study was designed to assess the potential of the underexploited buckwheat (*Fagopyrum esculentum*) farming in the UK.

## 2. Materials and Methods

### 2.1. General Phytochemical Analysis Methods and Chemicals

^1^H NMR, ^13^C NMR and 2D-NMR (COSY, NOESY, HMQC and HMBC) spectra were obtained on a JEOL 500 MHz instrument. Homonuclear ^1^H connectivities were determined by using the COSY experiment. One-bond ^1^H–^13^C connectivities were determined with HMQC while two- and three-bond ^1^H–^13^C connectivities were determined by HMBC experiments. Chemical shifts were reported in *δ* (ppm) using the solvent standard and coupling constants (*J*) were measured in Hz. The high-resolution mass spectroscopy instrument, Thermofisher LTQ Orbitrap XL (Thermofisher Scientific, city, UK), with an electrospray ionisation probe was used for an accurate mass measurement over the full mass range of m/z 50–2000. Nano-electrospray analyses were performed in positive ionisation mode by using NanoMate to deliver samples diluted into MeOH + 10% NH4OAc. The temperature was set at 200 °C, sheath gas flow of 2 units and capillary (ionizing) voltage at 1.4kV. The accurate mass measurements obtained from this system were far better than 3 ppm. Unless stated otherwise, all chemical standards and reagents are of Sigma-Aldrich Chemical Company Ltd (Dorset, Gillingham, UK).

### 2.2. Growing Condition

The seeds of common buckwheat were obtained from a local supermarket (Tesco) and planted in our experimental garden by mid-July. Apart from regular watering until the seeds germinate within two weeks and established in organically maintained well-drained soil, no other treatment was given. Being a very short seasonal plant, the plant flowers by the end of August, at which point the flowers and leaves were harvested, air-dried and powdered for analysis. The voucher specimen (no. SHM-BW-2018) was deposited in our Pharmacognosy Laboratories specimens’ collections.

### 2.3. Preparation of the Plant Material

A total of 5 L of methanol was added to the dried powdered leaves of buckwheat (240 g) in a flask, and left at room temperature for 3 days. After removal of the solvent under reduced pressure using a rotary evaporator, 35 g of the crude extract was obtained. The dried extract was used for antioxidant effect studies and isolation of rutin. For a quantitative determination of rutin, the powdered plant material (0.5 g) in triplicate was placed in a volumetric flask to make up a 100 mL of the methanol extract and sonicated for 15 min. Filtered extracts were then taken for the HPLC analysis. For comparative analysis, the dried flowers of buckwheat were also set up similarly.

### 2.4. HPLC Analysis

An Agilent 1200 series gradient HPLC system composed of a degasser (G1322A), a quaternary pump (G1322A), an auto sampler (G1329A), a thermostat column compartment (G1316A) maintained at 25 °C and a diode array detector (G1315D) was used. The concentrations of the standard rutin sample and plant extracts were injected (20 µL) onto a reverse phase column (Agilent – Eclipse Plus C18, 5 µm, 4.9 × 150 mm). The mobile phase was a mixture of water (A) and methanol (B). The composition of the mobile phase at a flow rate of 1 mL/min was rising from 10% to 90% B over a period of 50 min.

### 2.5. DPPH Radical Scavenging

The antioxidant activity of test samples was measured by using our established microtiter-based DPPH assay [15]. Briefly, a DPPH solution (0.1 mM, in methanol) was incubated with varying concentrations of test compounds for 20 min at room temperature, and the absorbance of the resulting solution was read at 550 nm against a blank using a Multiscan EX Reader (Thermo Labsystems, Altrincham, UK).

### 2.6. Measurement of Reducing Power

The reducing power of test agents was quantified by using the previously described method [16]. Briefly, 1 mL of the reaction mixture, containing different concentrations of samples in Dulbecco’s phosphate buffer saline (pH 7.0), was incubated with potassium ferricyanide (1%, w/v) at 50 °C for 20 min. Following the termination of the reaction by trichloroacetic acid solution (10%, w/v), ferric chloride (0.1%, w/v) was added to diluted (in distilled water) samples and the absorbance was measured at 700 nm. An increase in absorbance of the reaction mixture suggests a greater reducing power.

### 2.7. Determination of Fagopyrins Level

A quantitative estimation of fagopyrins level was made using the fluorescence method in microtiter plates. Briefly, 100 mg of the plant material was placed in 25 mL of a volumetric flask and 80% methanol (aqueous) was added. After 3 days under room temperature, the fluorescence associated with the fagopyrins content of the extract was measured using SpectraMax M5 Flurorimeter (Molecular Devices, Winnersh, Wokingham, UK) spectrophotometer at excitation 330 and emission 590. From the stock solution of 0.25 mg/mL hypericin, a correlation graph was constructed from which the level of fagoprins was determined.

### 2.8. Isolation of Rutin

The RediSep C18 gold column (100 g, Presearch, Hampshire, UK) attached to a Teledyne Isco flash chromatography system was used to isolate rutin as described previously [17,18]. Briefly, a linear gradient of methanol in water was established by starting from 10% methanol to 40% over a period of 15 min and then raising methanol to 90% at 20 min. The flow rate was maintained at 60 mL/min and the chromatogram was monitored by using dual wavelength of 214 and 254 nm. The leaves extract was subjected to this chromatographic condition to obtain pure rutin in just one chromatographic step, as with our previously published rutin isolation protocol.

## 3. Results and Discussion

### 3.1. The Rationale of Growing Buckwheat in European Countries Like the UK

Buckwheat is a traditional food in many parts of Russia and Northern China where, after hulling, the whole grain is boiled and eaten. Buckwheat is also used as a food in many other countries and served as the main ingredient for pancakes and noodles, among other things. While it is naturally gluten-free, compared with true grains, buckwheat is also acclaimed for its common mineral composition, such as zinc, copper, and manganese. Though not as big as it has been a century or so ago, buckwheat farming is still significant in the Far East and eastern European countries (Table 1).

Among the health benefits claimed in recent years, buckwheat’s green leaves have been shown to display a range of pharmacological effects through antioxidant mechanisms [9,10,11,12,13]. The flavonoids as a dominant phenolic constituent attributing to the antioxidant effect of buckwheat in the grain, seedling and the green leaves, have been reported [19,20,21]. As a single dominant active principle, this antioxidant effect has been shown to correlate with the high content of rutin in common buckwheat [22,23]. In this connection, the potential of the plant grown in the UK is assessed in this communication.

Buckwheat is a fast-growing plant that can be farmed in countries with a short summer season. In just over a month starting from July, the plant completes the whole cycle of life from sowing to harvest at the flowering stage. It is for this reason that this semi-succulent fast-growing plant is often recommended as a ground cover, green manure and for attracting insects including honeybees.

### 3.2. Antioxidant and Reducing Power of Buckwheat Leaves Extract

**Figure 2:**
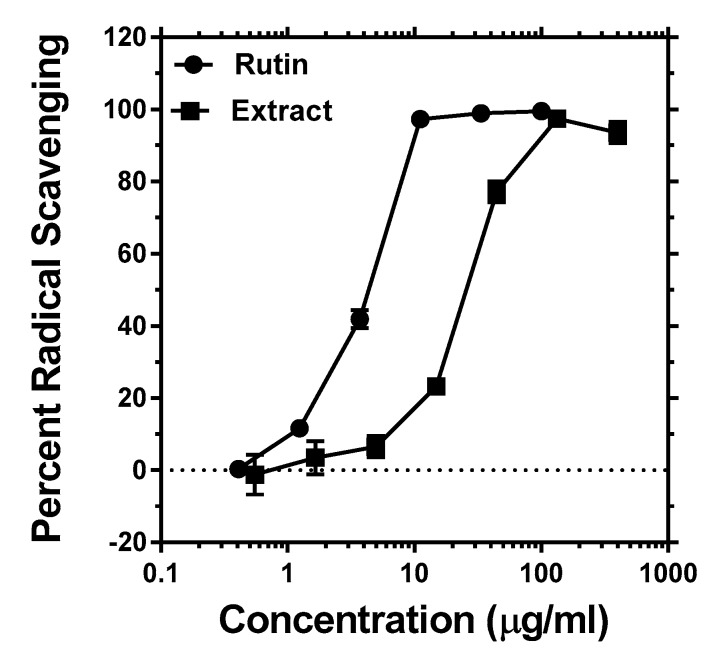
Radical scavenging profile of buckwheat leaves extract and rutin.

The antioxidant activity of the methanolic extract of the plant samples was tested by using the DPPH radical scavenging assay. Given that rutin is the known bioactive component of the buckwheat plant and that it is commonly employed as a positive control for antioxidant assays in medicinal plants analysis [24,25,26], its effect is also assessed herein. As shown in Figure 2, a potent concentration-dependent radical scavenging effect was demonstrated both for the crude extract and the positive control, rutin. The calculated IC_50_ values obtained from four separate experiments (Table 2) further revealed that the positive control was about five times more potent than the crude extract. Given that crude plants extracts are mixtures of so many compounds of organic and inorganic nature, the observed antioxidant activity of the crude extract should be considered very potent.

**Figure 3:**
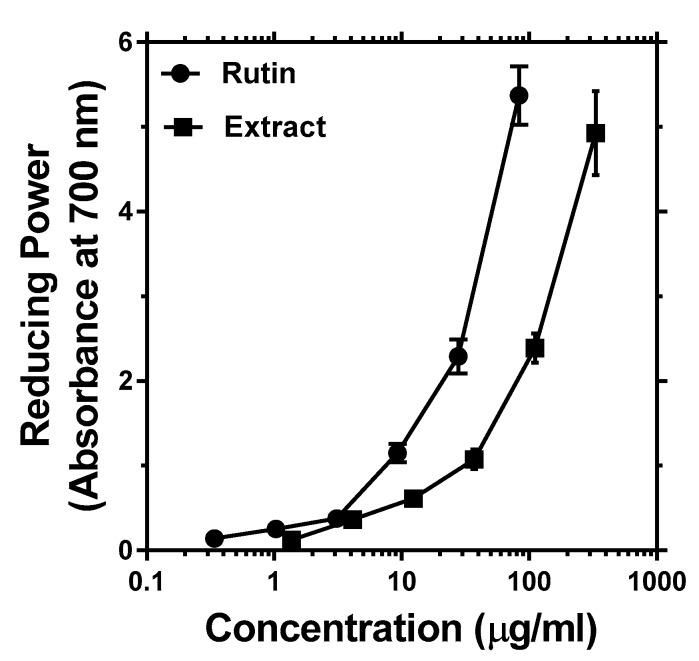
Reducing power of methanolic extract of buckwheat leaves and rutin based on measurement of Fe^3+^–Fe^2+^ transformation.

Through donation of hydrogen atom and breaking the free radical chain, many antioxidant compounds also act as reductones, as we have shown for a range of natural products [15,16,27]. The reductive capacity of buckwheat in comparison with rutin is shown in Figure 3. On the basis of the Fe^3+^–Fe^2+^ transformation, the concentration-dependent reducing power is evident. Hence, the antioxidant and reducing power of the extract and its main component, rutin could be confirmed for buckwheat green.

### 3.3. Quantitative Determination of Rutin in Buckwheat Leaves

**Figure 4:**
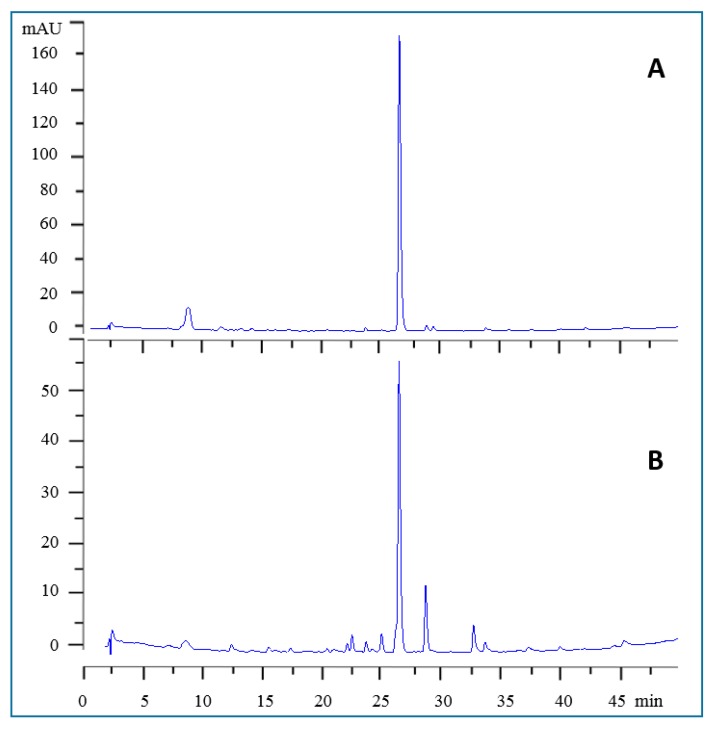
HPLC Chromatograms of the leaves (top panel, A) and flowers (lower panel, B) extracts of buckwheat extracts. The HPLC analysis was based on 5 mg/mL solution of crude extracts in methanol as described in the material and methods. The chromatograms show the corresponding peaks detected at 280 nm. Notice the major peak in both extracts as that of rutin (retention time ~ 26 min) and the flowers extract has more components than the leaves.

Given that buckwheat has been considered as one of the most useful commercial sources of rutin for a high yield extraction [13], the content of this bioactive compound was analysed by HPLC. While crude plant extracts display complex HPLC profiles due to the presence of numerous secondary metabolites, the chromatogram (Figure 4) for buckwheat leaves almost exclusively shows rutin as one principal phenolic component. On the other hand, the flowers extract contains additional components, although rutin is still the major compound (Figure 4). For quantitative analysis, the HPLC response taken as the peak area under curve was plotted against rutin concentrations. A straight-line equation with *r*^2^ value extremely close to one (0.9999) was achieved. The content of the rutin analysis using GraphPad software (Prism) from three independent experiments is shown in Table 3. Hence, the external standard-based analysis of the rutin revealed that the leaves are packed-full of this bioactive compound with the yield of 3.4 g/100 g (on dry weight basis (DW)). For comparative purposes, the HPLC profile of the flowers extract is also shown in Figure 4, and while rutin is the predominant compound, the flowers’ extract appears to be a more complex mixture. In fact, the rutin content of the flowers was about four times less than those of the leaves on dry weight basis (Table 3).

### 3.4. Isolation and Characterization of Rutin from Buckwheat Leaves

Furthermore, the major component of the leaves extract was isolated using Teledyne Isco flash chromatography system and identified based on spectroscopic analysis, primarily by 1D (^1^H, ^13^C and DEPT) and 2D NMR (HMQC, HMBC and COSY) and mass spectrometry. The compound showed [M + H]^+^, C_27_H_30_O_16_ plus H, at m/z 611.1603, [M + Na]^+^ at 633.1420; and [2M + Na]^+^ at 1243.2968. These together with the NMR data, which were all in good agreement with our own previous reports [18,28], allowed the identification of the compound as rutin.

### 3.5. Fagopyrins Content Analysis of Buckwheat Leaves

Despite the acclaimed health potential of buckwheat greens, they also contain phototoxic naftodianthrones called fagopyrins (Figure 1). Structurally, these are compounds similar with hypericin but incorporate nitrogen in their structures. Phototoxic effects caused by fagopyrins following exposure to sunshine is known as fagopyrism [12,29]. Hence, consuming large amounts of buckwheat leaves has been associated with induction of dermatitis, breathlessness, fainting, and hair loss [30]. The phototoxicity of fagopyrins is similar with that of hypericin, and toxicity in rodents at doses of 2.5–3 mg/kg [31] was reported, while toxic doses in humans remain to be established. A number of studies on fagopyrin contents of the leaves have been published and in the common buckwheat herb, 1.6–4.8 mg/g was reported, while the leaves are known to contain 0.3–2.3 mg/g [30,32,33,34]. On the other hand, flowers have been shown to have a higher fagopyrin content of up to 20.8 mg/g dry weight [30]. For a reasonable exploitation of rutin in high yield or potential nutraceutical applications, the leaves that can be obtained in high yield but also with less fagopyrin content are ideal. The fagopyrin content detected in the leaves in this investigation is very low (Table 3) but whether this level could be associated with toxic effect in humans still remains to be established. The far inferior (1.7-times) fagopyrin content in the leaves compared to that in the flowers along with the rutin content analysis data (Table 3) suggest that the leaves have better potential for further development as functional food or large scale production of rutin.

### 3.6. General Summary and Conclusion

Rutin is a multifunctional natural product that has been shown to display numerous pharmacological activities. Extensive review articles on the pharmacology of rutin have been published in recent years, including from our own laboratories as potential therapy for diabetes [35], Alzheimer’s disease [36], and inflammatory brown disease [37]. In view of commercial exploitation of this bioactive compound, plants such as *Moringa stenopetala* have been subjected to extensive research in our laboratories and shown their promise as valuable natural source of rutin [28,38]. Perhaps the highest yield of rutin as a commercial source however is buckwheat, with two species, *F. esculentum* and *F. tataricum*, being the most studied. The aerial parts of these plants, such as the flowers and leaves, have been routinely shown to contain from about 2 to 10 g/100 g of rutin on DW basis [39,40,41]. As with many natural products, the concentration of rutin in buckwheat could vary based on a number of factors such as genetic or cultivar variability [42], plant parts such as leaves versus flowers [43] and growing conditions or environmental factors, including exposure to UV light [41,44]. In view of these variations, the present study was designed to ascertain whether buckwheat grown in the UK could have a potential as a source of rutin or bioactive buckwheat green extract. The immersive yield of rutin as 3417 mg/100 g DW from the leaves is a good starting point to initiate a further study on exploitation of this fast growing and rather underutilized plant in Europe. In this connection, buckwheat leaves grown in Eastern European countries under different growing conditions have been shown to contain rutin in the range of between 2170 mg/100 g and 3430 mg/100 g [11]. The observed variation in rutin content between leaves and flowers was also in agreement with previous studies [10].

Even though buckwheat flowers are known for a high yield of rutin [43], functional foods from buckwheat have been known for allergic reactions, including hypersensitivity such as asthma and gastrointestinal disorders [45]. The main problem of the buckwheat aerial parts, particularly the flowers, are however the fagopyrism or phototoxicity. It remains to be established whether the level of fagopyrins detected in the leaves of common buckwheat plant grown in the UK is a health risk to humans when consumed without processing. Method development for effective removal of fagopyrins with implication for functional food development, however, has already been suggested [12] and further studies in this field will be vital to realize the full potential of the plant. In the meantime, this case study could open the way to fruitful buckwheat cultivation in the UK, provided that further extensive research is performed.

## Figures and Tables

**Table 1:** Quantity of global buckwheat production in the year 2017 *.

Country	Tons	Remark	Ranking
Russian Federation	1524280		1
China	1447292		2
Ukraine	180440		3
France	127406	IM	4
Kazakhstan	120379		5
Poland	113113		6
United States of America	76362	IM	7
Brazil	64500		8
Lithuania	53221		9
Japan	34400		10
United Republic of Tanzania	21663	IM	11
Belarus	18010		12
Latvia	17100		13
Nepal	12039		14
Bhutan	3480		15
Estonia	3385		16
Slovenia	2909		17
Czechia	2365	IM	18
Republic of Korea	1633	IM	19
Bosnia and Herzegovina	1187		20
Croatia	624		21
Hungary	500		22
Canada	463	IM	23
Slovakia	367		24
South Africa	232	IM	25
Republic of Moldova	192	IM	26
Georgia	112	IM	27
Kyrgyzstan	94		28

IM = FAO data based on imputation methodology; * The FAO data [14] on production quantities in tons and ranking order for producing countries are shown.

**Table 2:** Antioxidant effect of buckwheat leaves extract and rutin.

	IC_50_ (μg/mL) *
Crude extract	20.00 ± 2.67
Rutin	3.93 ± 0.52

* Mean and standard error of mean (SEM) values obtained from 4 separate experiments in the DPPH assay are shown.

**Table 3:** Rutin and fagopyrins contents (mg/100 g DW) of buckwheat leaves and flowers *.

-	Leaves	Flowers
Rutin	3417 ± 122	822 ± 162
Fagopyrins	19 ± 2	32 ± 4

* Mean and SEM values from 4 separate experiments.

## References

[1] Ahmed A., Khalid N., Ahmad A., Abbasi N.A., Latif M.S.Z., Randhawa M.A. (2014). Phytochemicals and biofunctional properties of buckwheat: a review. J. Agric. Sci..

[2] Li S.Q., Zhang Q.H. (2001). Advances in the development of functional foods from buckwheat. Crit. Rev. Food Sci. Nutr..

[3] Wijngaard H.H., Arendt E.K. (2006). Buckwheat. Cereal Chem..

[4] Guo Y.Z., Chen Q.F., Yang L.Y., Huang Y.H. (2007). Analyses of the seed protein contents on the cultivated and wild buckwheat *Fagopyrum esculentum* resources. Genet. Resour. Crop Evol..

[5] Eggum B.O., Kreft I., Javornik B. (1980). Chemical composition and protein quality of buckwheat (*Fagopyrum esculentum* Moench). Plant Foods Hum. Nutr..

[6] Tahir I., Farooq S. (1985). Grain composition in some buckwheat cultivars (*Fagopyrum* spp.) with particular reference to protein fractions. Plant Foods Hum. Nutr..

[7] Skrabanja V., Liljeberg Elmståhl H.G., Kreft I., Björck I.M. (2001). Nutritional properties of starch in buckwheat products: studies in vitro and in vivo. J. Agric. Food Chem..

[8] Benvenuti M.N., Giuliotti L., Pasqua C., Gatta D., Bagliacca M. (2012). Buckwheat bran (*Fagopyrum esculentum*) as partial replacement of corn and soybean meal in the laying hen diet. Ital. J. Anim. Sci..

[9] Prakash S., Yadav K. (2016). Buckwheat (*Fagopyrum esculentum*) as a functional food: A Nutraceutical Pseudocereal. Int. J. Curr. Trend. Pharmacobiol. Med. Sci..

[10] Paulickova L., Vyb’alova K., Holasova M., Fiedlerova V., Vavreinova S. Buckwheat as functional food. Proceedings of the 9th International Symposium on Buckwheat.

[11] Dražić S., Glamočlija Đ., Ristić M., Dolijanović Ž., Dražić M., Pavlović S., Jaramaz M., Jaramaz D. (2016). Effect of environment of the rutin content in leaves of Fagopyrum esculentum Moench. Plant Soil Environ..

[12] Kreft S., Jane D. (2013). The content of fagopyrin and polyphenols in common and tartary buckwheat sprouts. Acta Pharm..

[13] Humphreys F.R. (1964). The occurrence and industrial production of rutin in Southeastern Australia. Econ. Bot..

[14] FAOSTAT (Statistics Division of Food and Agriculture Organization of the United Nations) Buckwheat. http://www.fao.org/faostat/en/#data/QC.

[15] Habtemariam S., Jackson C. (2007). Antioxidant and cytoprotective activity of leaves of *Peltiphyllum peltatum* (Torr.). Engl. Food Chem..

[16] Habtemariam S. (2007). Antioxidant activity of knipholone anthrone. Food Chem..

[17] Habtemariam S., Varghese G.K. (2015). Extractability of Rutin in Herbal Tea Preparations of *Moringa stenopetala* Leaves. Beverages.

[18] Habtemariam S. (2015). Investigation into the antioxidant and antidiabetic potential of Moringa stenopetala: Identification of the active principles. Nat. Prod. Commun..

[19] Sytar O., Gabr A.M., Smetanska I., Kosyan A. (2011). Pigments, phenolic contents and antioxidant activity of buckwheat seedlings under in vivo and in vitro conditions. Climate Change: Challenges and Opportunities in Agriculture, Proceedings of the AGRISAFE Final Conference, Budapest, Hungary, 21–23 March 2011.

[20] Alvarez-Jubete L., Wijngaard H., Arendt E.K., Gallagher E. (2010). Polyphenol composition and in vitro antioxidant activity of amaranth, quinoa buckwheat and wheat as affected by sprouting and baking. Food Chem..

[21] Zielinski H., Michalska A., Amigo-Benavent M., del Castillo M.D., Piskula M.K. (2009). Changes in protein quality and antioxidant properties of buckwheat seeds and groats induced by roasting. J. Agric. Food Chem..

[22] Kalinova J., Triska J., Vrchotova N. (2006). Distribution of Vitamin E, squalene, epicatechin, and rutin in common buckwheat plants (*Fagopyrum esculentum* Moench). J. Agric. Food Chem..

[23] Ohara T., Ohinata H., Muramatsu N., Matsuhashi T. (1989). Determination of rutin in buckwheat foods by high performance liquid chromatography. Nippon. Shokuhin Kogyo Gakkaishi.

[24] Habtemariam S., Varghese G.K. (2017). Antioxidant, anti-alpha-glucosidase and pancreatic beta-cell protective effects of methanolic extract of *Ensete superbum* Cheesm seeds. Asian Pac. J. Trop. Biomed..

[25] Habtemariam S., Cowley R.A. (2012). Antioxidant and anti-α-glucosidase compounds from the rhizome of *Peltiphyllum peltatum* (Torr.). Engl. Phytother. Res..

[26] Roselli M., Lentini G., Habtemariam S. (2012). Phytochemical, antioxidant and anti-alpha-glucosidase activity evaluations of *Bergenia cordifolia*. Phytother. Res..

[27] Habtemariam S. (2008). Activity-guided isolation and identification of free radical-scavenging components from ethanolic extract of boneset (eaves of *Eupatorium perfoliatum*). Nat. Prod. Commun..

[28] Habtemariam S. (2017). The African and Arabian Moringa Species: Chemistry, Bioactivity and Therapeutic Applications.

[29] Benković E.T., Kreft S. (2015). Fagopyrins and Protofagopyrins: Detection, analysis, and potential phototoxicity in buckwheat. J. Agric. Food Chem..

[30] Stojilkovski K., Kočevar Glavač N., Kreft S., Kreft I. (2013). Fagopyrin and flavonoids content in common, tartary, and cymosum buckwheat. J. Food Comp. Anal..

[31] Theurer C., Gruetzner K.I., Freeman S.J., Koetter U. (1997). In vitro phototoxicity of hypericin, fagopyrin rich, and fagopyrin free buckwheat herb extracts. Pharm. Pharmacol. Lett..

[32] Eguchi K., Anase T., Osuga H. (2009). Development of a high-performance liquid chromatography method to determine the fagopyrin content of tartary buckwheat (*Fagopyrum tataricum* Gaertn.) and common buckwheat (*F. esculentum* Moench). Plant Prod. Sci..

[33] Hinneburg I., Neubert R.H.H. (2005). Influence of extraction parameters on the phytochemical characteristics of extracts from buckwheat (*Fagopyrum esculentum*) herb. J. Agric. Food Chem..

[34] Glavač N.K., Stojilkovski K., Kreft S., Park C.H., Kreft I. (2017). Determination of fagopyrins, rutin, and quercetin in Tartary buckwheat products. LWT Food Sci. Technol..

[35] Habtemariam S., Lentini G. (2015). The therapeutic potential of rutin for diabetes: An update. Mini Rev. Med. Chem..

[36] Habtemariam S. (2016). Rutin as a natural therapy for Alzheimer’s disease: Insights into its mechanisms of action. Curr. Med. Chem..

[37] Habtemariam S., Belai A. (2018). Natural Therapies of the inflammatory bowel disease: The case of rutin and its aglycone, quercetin. Mini Rev. Med. Chem..

[38] Habtemariam S. (2016). The African *Moringa* is to change the lives of millions in Ethiopia and far beyond. Asian Pac. J. Trop. Biomed..

[39] Kalinova J., Dadakova E. Varietal differences of rutin in common buckwheat (*Fagopyrum esculentum* Moench) determined by micellar electrokinetic capillary chromatography. Proceedings of the 9th International Symposium on Buckwheat.

[40] Fabjan N., Rode N., Košir I.J., Wang Z., Zhang Z., Kreft I. (2003). Tartary buckwheat (Fagopyrum tataricum Gaertn.) as a source of dietary rutin and quercitrin. J. Agric. Food Chem..

[41] Suzuki T., Honda Y., Mukasa Y. (2005). Effects of UV-B radiation, cold and desiccation stress on rutin concentration and rutin glucosidase activity in tartary buckwheat (*Fagopyrum tataricum*) leaves. Plant Sci..

[42] Ohsawa R., Tsutsumi T., Matano T., Ujihara A. (1995). Improvement of rutin content in buckwheat flour. Current Advances in Buckwheat Research, Proceedings of Sixth International Symposium on Buckwheat at Ina, Nagano, Japan, 24–29 August 1995.

[43] Hagels H. (1999). *Fagopyrum esculentum* Moench. Chemical review. Zbornik BFUL.

[44] Harborne J.B., Williams C.A. (2000). Advances in flavonoid research since 1992. Phytochemistry.

[45] Wieslander G., Norbäck D. (2001). Buckwheat allergy. Allergy.

